# 3D analysis of microvasculature in murine liver fibrosis models using synchrotron radiation-based microtomography

**DOI:** 10.1007/s10456-020-09751-9

**Published:** 2020-10-10

**Authors:** Willi L. Wagner, Sonja Föhst, Jessica Hock, Yong Ook Kim, Yury Popov, Detlef Schuppan, Katja Schladitz, Claudia Redenbach, Maximilian Ackermann

**Affiliations:** 1 Department of Diagnostic and Interventional Radiology, University of Heidelberg, Heidelberg, Germany; 2 Translational Lung Research Center, Member of the German Center for Lung Research, University of Heidelberg, Heidelberg, Germany; 3 Mathematics Department, Technische Universität Kaiserslautern, Kaiserslautern, Germany; 4 Institute of Functional and Clinical Anatomy, University Medical Center of the Johannes Gutenberg-University Mainz, Johann-Joachim-Becher-Weg 13, 55128 Mainz, Germany; 5 Institute of Translational Immunology and Research Center for Immune Therapy (FZI), University Medical Center of the Johannes Gutenberg-University, Mainz, Germany; 6 Division of Gastroenterology, Beth Israel Deaconess Medical Center, Harvard Medical School, Boston, MA USA; 7 Image Processing Department, Fraunhofer ITWM, Kaiserslautern, Germany; 8 Institute of Pathology and Molecular Pathology, Helios University Clinic Wuppertal, University of Witten-Herdecke, Wuppertal, Germany

**Keywords:** Cirrhosis, Angiogenesis, Intussusceptive angiogenesis, Image analysis, Synchrotron radiation microcomputed tomography

## Abstract

Cirrhosis describes the development of excess fibrous tissue around regenerative nodules in response to chronic liver injury and usually leads to irreversible organ damage and end-stage liver disease. During the development of cirrhosis, the formation of collagenous scar tissue is paralleled by a reorganization and remodeling of the hepatic vascular system. To date, macrovascular remodeling in various cirrhosis models has been examined using three-dimensional (3D) imaging modalities, while microvascular changes have been studied mainly by two-dimensional (2D) light microscopic and electron microscopic imaging. Here, we report on the application of high-resolution 3D synchrotron radiation-based microtomography (SRμCT) for the study of the sinusoidal and capillary blood vessel system in three murine models of advanced parenchymal and biliary hepatic fibrosis. SRμCT facilitates the characterization of microvascular architecture and identifies features of intussusceptive angiogenesis in progressive liver fibrosis in a non-destructive 3D manner.

## Introduction

Cirrhosis is caused by an excess deposition of collagenous connective tissue in an abnormal wound healing response to continuous liver injury [1–3]. In the process of ongoing fibrogenesis and increasing fibrosis, the vascular system is adversely reorganized to affect the hepatic circulation on the macroscopic and the microscopic scale, leading to severe clinical complications such as portal hypertension and functional liver failure. On the micron scale, sharp bends, abnormal branching, and an increased tortuosity of vessels were observed by deep tissue microscopy (DTM) [4]. These microvascular alterations likely cause an aggravation of portal hypertension during hepatic fibrosis [5]. On the micron level, fibrotic liver tissue and the sinusoidal vascular system are conventionally investigated by light microscopy of histopathological sections [6] or by ultrastructural analysis using electron microscopy [7]. While this allows for a 2D evaluation of the degree of fibrosis and morphological aspects of the capillary system, crucial parameters characterizing the microvascular morphology and architecture can only be derived from 3D imaging. 3D image acquisition methods such as computed tomography and magnetic resonance imaging have been established as a valuable tool to investigate the hepatic vascular system during cirrhosis on a macroscopic level [8, 9]. The 3D assessment of the sinusoidal vascular system during liver fibrosis, especially in mouse models, imposes advanced technical requirements to the applied imaging systems. Temporal and spatial resolution of state-of-the-art microcomputed tomography scanners allow for qualitative assessment of the hepatic macrovasculature in vivo [10] and quantitative assessment of the hepatic macrovasculature ex vivo [4]. Sinusoids in small rodents typically have a diameter of 5–9 μm [11], requiring an imaging approach on the submicron scale [4]. However, the spatial resolution of in vivo μCT scans is limited to 35 μm voxel edge length [6]. With the use of synchrotron radiation-based microtomography (SRμCT), by providing a high photon flux and phase-sensitive imaging protocols, spatial resolutions of 10.9 to 3.5 μm isometric voxel sizes were reached in the assessment of liver fibrosis [12–17]. While a detailed depiction and characterization of macrovessels and smaller intrahepatic arterioles and venules was achieved, the sinusoidal vascular bed remained beyond the spatial resolution capacities in previous studies.

To compensate for the loss in number and structure of blood vessels, the formation of new blood vessels is induced in a process known as angiogenesis. Both in human and murine liver samples, a correlation between the number of newly formed blood vessels and the progression of liver fibrosis could be observed [5, 6]. Two mechanisms of angiogenesis can be distinguished [18–22]: In sprouting angiogenesis, new branches diverge from pre-existing blood vessels. In intussusceptive angiogenesis, intussusceptive pillars (IP), small tissue bridges, form within a pre-existing blood vessel. By growth of the pillar, the capillary is split into two new vessels. Intussusceptive angiogenesis is a morphogenetic process which is frequently seen in physiological [18] and pathological conditions [21, 22].

In this brief report, we apply high-resolution SRμCT at a voxel edge length of 325 nm for 3D visualization and quantitative assessment of the sinusoidal blood vessel system in three different murine models of fibrosis using microvascular corrosion casting [4]. The small volume of interest does not allow for a segmentation of the entire hepatic vascular tree. Hence, we concentrate on the evaluation of local geometric characteristics to depict features of microvascular remodeling during hepatic fibrosis. Additionally, intussusceptive angiogenesis is assessed by evaluating the frequency of IP in 3D. A characterization of the changes in microvascular morphology and architecture during progressive hepatic fibrosis may improve our understanding of the natural history and pathophysiology of cirrhosis and thereby improve treatment and management of cirrhotic patients.

## Materials and methods

### Animals

Hepatotoxin-induced liver fibrosiss was induced in 7–8-week-old C57B6 mice (Charles River, Munich) by chronic injections of thioacetamide (TAA, Sigma-Aldrich, Munich) or carbon tetrachloride (CCl4, Sigma-Aldrich, Munich) in incremental doses to produce a robust and reproducible precirrhotic collagen accumulation (stage 2–3 out of 4), as described by us [3]. Briefly, CCl4 was given in mineral oil via oral gavage three times a week for 6 weeks according to an escalating dose protocol (first dose, 0.875 ml/kg; week 1–3, 1.75 ml/kg; week 4–6, 2.5 ml/kg). Alternatively, fibrosis was induced by escalating the intraperitoneal dosage of TAA for 6 weeks (first dose, 100 mg/kg, week 1–2, 200 mg/kg; week 3–4, 300 mg/kg; week 4–6, 400 mg/kg). Mice were sacrificed three days after the last CCl4 or TAA application. In both cases, a second group (regression) was treated for 6 weeks and allowed to (partly) recover during a subsequent period of 3 weeks without further treatment. Notably, this regression group displays normalized inflammation parameters and an improved scar tissue microarchitecture with dissipated collagen bundles, but shows only minimal biochemical fibrosis regression [3]. Mice of the control group received vehicle injections only.

ABCB4−/− FVB mice were bred at Institute of Translational Immunology and Research Center for Immune Therapy (FZI), University Medical Center of the Johannes Gutenberg-University [22]. Due to the manipulation of the ABCB4 gene, the protein MDR2 is missing. These mice spontaneously develop progressive biliary liver disease and advanced fibrosis, resembling fibrosis stage 2–4 human primary sclerosing cholangitis, between 8 and 12 weeks of age. They were sacrificed at an age of 10–12 weeks. The control group was formed by wildtype FVB littermates not showing any fibrosis. The care of the animals was consistent with legal guidelines and was approved by the Institutional Animal Care and Use Committee of Rhineland-Palatinate (Koblenz, Germany).

### Vascular corrosion casting

After systemic heparinization with 2000 U/kg heparin intraperitoneally, mice were laparotomized under deep anesthesia. The portal vein and the aorta were cannulated with an olive-tipped cannula and perfused with 10 ml saline at 37 °C. The vascular network was fixed with 2.5% phosphate buffered glutaraldehyde (pH 7.40, 300 mosmol) over a period of 2–5 min. The resin was prepared under a hood by mixing 50 g of the pre-polymerized polyurethane-based PU4ii (vasQtec, Zurich, Switzerland), 30 g of ethylmethylketone (Merck, Darmstadt, Germany), and 50 mg dye (vasQtec, Zurich, Switzerland) with a magnetic stirrer. The casting medium was injected by constant pressure under normal control flow rate. Samples were placed for 5 h in an incubator or waterbath at 40 °C until complete polymerization of the resin. The liver was dissected and macerated in 5% potassium hydroxide (Merck, Darmstadt, Germany) at 40 °C for 2–3 days. Macerated samples were cleaned from potassium hydroxide with distilled water and frozen in fresh distilled water. The specimens were osmicated with 1% osmium tetroxide and were imaged using SRμCT.

### Synchrotron radiation-based microtomography (SRμCT)

**Fig. 1:**
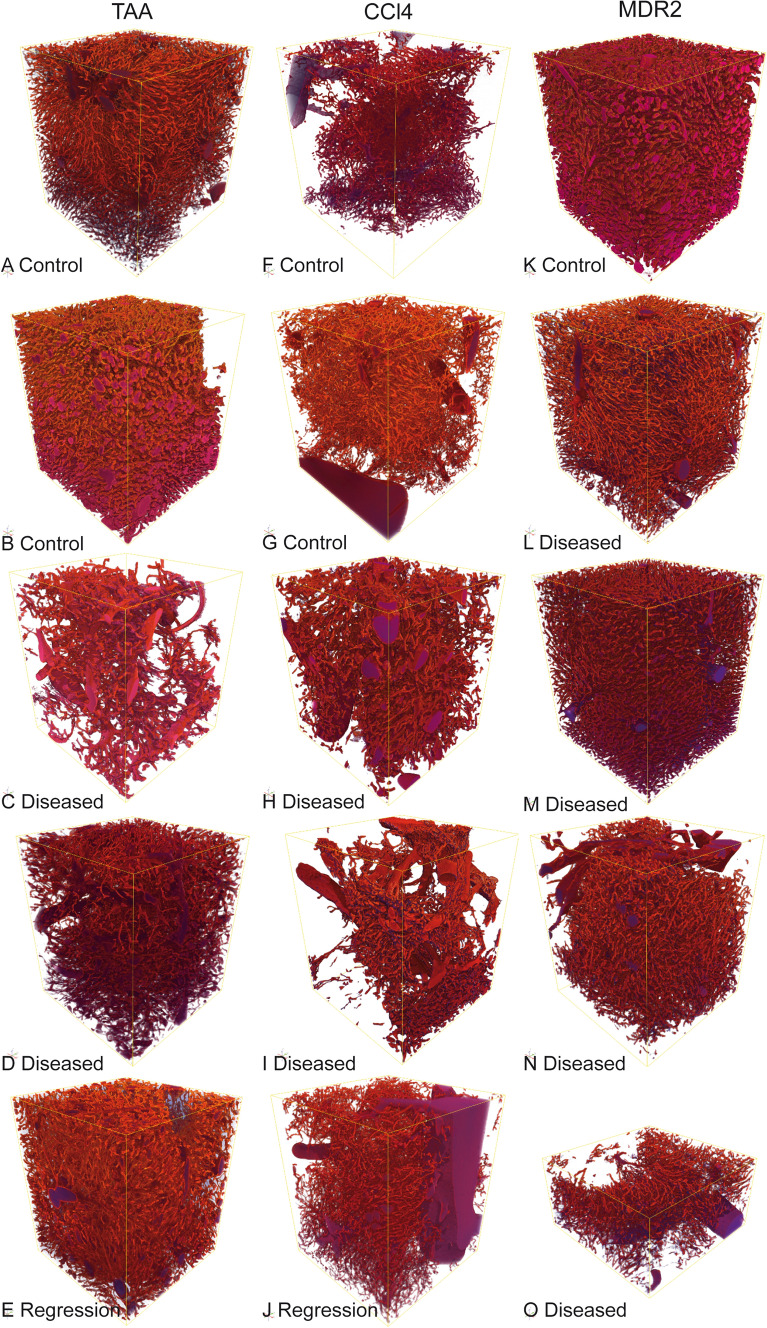
3D volume renderings of all evaluated scans with isometric voxel size of 325 nm

**Fig. 2:**
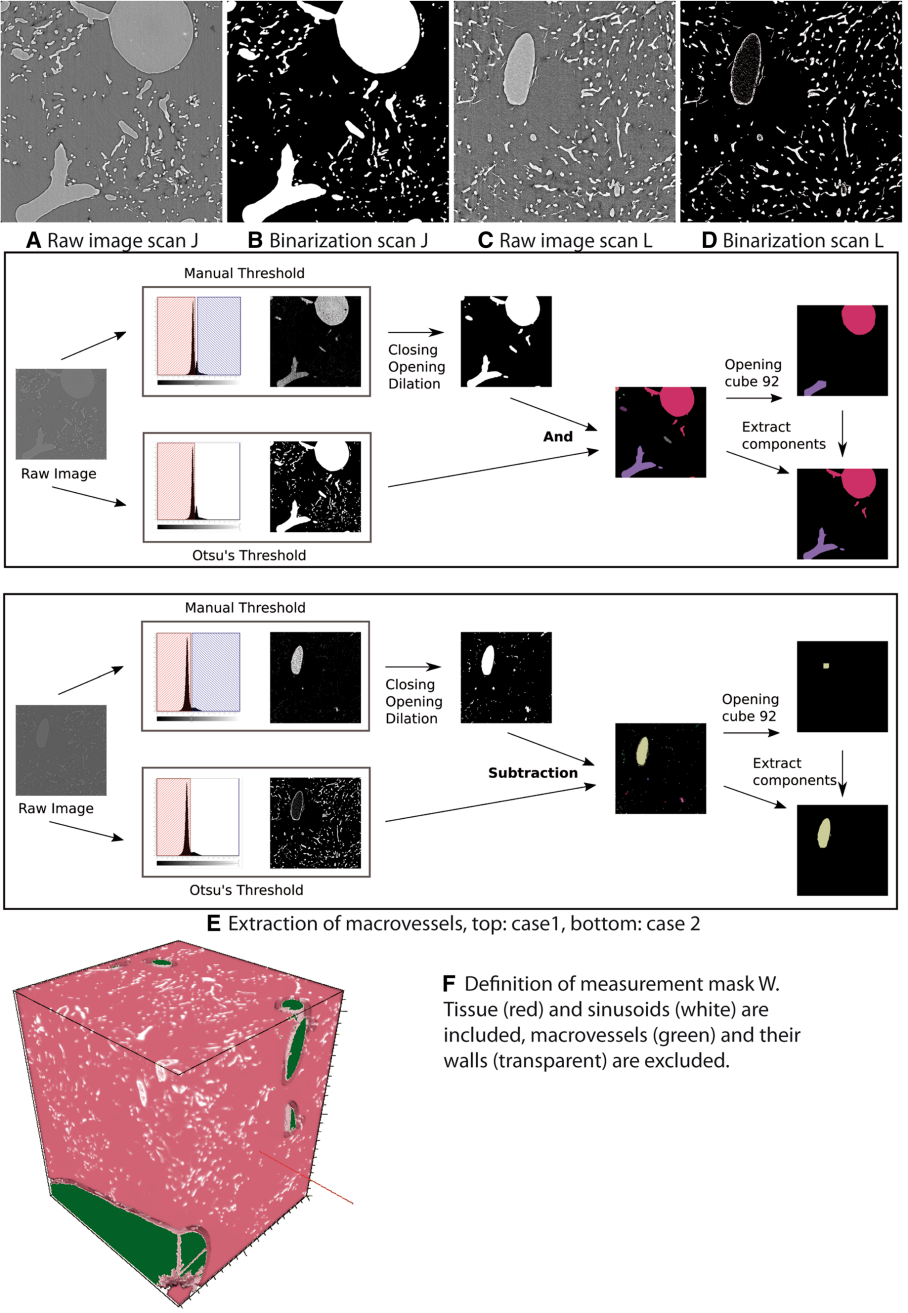
**a**–**d** 2D slices of 3D raw image and binarization of two scans. 1810 × 1810 voxels, voxel size 325 nm, physical size about 590 μm × 590 μm. **e** Workflow for the extraction of vessels with diameter larger than 30 μm. **f** Visualization of observation window W

Samples were scanned with the TOMCAT Beamline at the Swiss Light Source of the Paul Scherrer Institute (Villigen, Switzerland). The X-ray wavelength was 0.1 nm, corresponding to an energy of 12.398 keV. The monochromatic X-ray beam (Δ*E*/*E* = 0.014%) was tailored by a slits system to a profile of 1.4 mm^2^. After penetration of the sample, X-rays were converted into visible light by a Ce-doped YAG scintillator screen (Crismatec Saint-Gobain, Nemours, France). Projection images were magnified by diffraction-limited microscope optics and digitized by a high-resolution CCD camera (Photonic Science, East Sussex, UK). For each measurement, 1001 projections were acquired along with dark and periodic flat field images at an integration time of 4 s each without binning. Data were postprocessed and rearranged into flat field-corrected sinograms online. After reconstruction, the scans had a volume of 2560 × 2560 × 2160 voxels with a voxel edge length of 325 nm, representing a physical volume of 832 μm × 832 μm × 702 μm (less than 0.1% of the entire liver). Volumes of interest were randomly selected with all positions within the liver parenchyma having the same chance of being imaged. 17 liver specimens were recorded. For each specimen, one volume of interest was scanned. Volume renderings of all scans are shown in Fig. 1. Examples of 2D sectional images can be found in Fig. 2. Two samples with obvious artificial extravasation of the casting medium had to be excluded from the analysis.

#### Image processing

Image processing was performed using the software package MAVI, version 1.5.3 [23]. Details regarding the image processing algorithms can be found in [24].

#### Segmentation of the blood vessel system

To denoise the image, a median filter with filter mask of size 7 × 7 × 7 was applied. Afterwards, the blood vessel system was segmented by global thresholding. The threshold determined by Otsu’s method [25] yields an accurate segmentation of the capillary vessels. Postprocessing is required as Otsu’s method assigns the interior of macrovessels to the foreground in some of the scans (case 1, Fig. 2b), while the vessels’ interior is segmented as background (case 2, Fig. 2d) in the remaining scans.

In a first postprocessing step, holes inside the vessel structure were removed. Therefore, connected components of the background were labeled. Each connected component completely included in the vessel system was assigned to the vessels. Note that this procedure is not sufficient for filling the macrovessels in the second case described above. Subsequently, an opening with a cubical structuring element with three voxels edge length is applied to remove small noise components.

#### Extraction of macrovessels

Our study focuses on the system of sinusoidal and capillary vessels—for simplicity, we will only use the term sinusoids in the following—i.e., blood vessels with a diameter smaller than 30 μm [26, 27]. Larger vessels are excluded from the analysis by the procedure shown in Fig. 2e). In case 1, Otsu’s threshold was reduced manually until most of the vessel interior was assigned to the foreground. Afterwards, a threshold from above was chosen to assign bright sinusoid voxels to the background. In case 2, increasing the threshold manually resulted in a segmentation of macrovessels, only.

In both cases, additional morphological operations were applied to smooth the results: a closing with cubical structuring element of size 4, an opening with cubical structuring element of size 6, and dilation of the foreground using a cubical structuring element (size 6 in case 1 and size 20 in case 2). In case 1, the Otsu binarization was subtracted from the smoothed result. In case 2, the Otsu binarization and the smoothed image were combined by an and- operation. Finally, the connected components of the foreground were detected by a labeling algorithm. An opening with a cube of size 92 (= 29.9 μm) removed each connected component always having a size less or equal to 30 μm. The remaining labels indicate the connected components representing macrovessels.

### Analysis

Characteristics for the 3D microvascular structure are derived under the assumption that the sinusoidal system forms a random closed set [24]. Due to the small volumes observed in the SRμCT images, we additionally assume that—after removal of the macrovessels—the sinusoidal structure is stationary. As a consequence, statistics such as the vessel content or vessel diameter may be averaged over the full scan.

#### Microvascular volume and length

For a stationary random set *X* observed in a window *W*, the volume density is defined as the expected fraction of *W* that is contained in *X*. Here, *X* is the sinusoidal system. A default choice for *W* is the full volume observed in the SRμCT scan. However, Fig. 1 indicates that the amount of macrovessels contained in the different specimens differs significantly. Hence, macrovessels are excluded from the observation window *W*. Furthermore, the volume renderings indicate that regions surrounding the macrovessels do not contain any sinusoids. This can be explained by the fact that sinusoids cannot be contained in the macrovessels’ walls. Consequently, the system of macrovessels is dilated with a ball of radius 15 µm and excluded from the window, see Fig. 2f). Hence, the measurement mask *W* can be interpreted as that region in the scan that belongs to capillary liver tissue and the volume fraction is estimated by $V_{{\text{V}}} = \frac{V\left( X \right)}{{V\left( W \right)}},$ where *V* denotes volume. To assess the amount of sinusoids in the reference volume W, the specific microvascular length, i.e., the mean total sinusoid length per unit volume, is computed as

$L_{{\text{V}}} = \frac{M\left( X \right)}{{V\left( W \right)\pi \left( {1 - V_{{\text{V}}} } \right)}},$ where *M*(*X*) is the integral of mean curvature of the sinusoidal system.

#### Microvascular diameter

Local thickness of sinusoids is determined by the granulometry distribution function. It assigns to each point of X the diameter of the largest ball completely contained in X and covering that point [24]. By plotting the histogram of such diameters for all sinusoid voxels, a volume weighted diameter distribution—the granulometry curve—of the sinusoids is obtained. Due to discretisation errors, small balls with a diameter of only one or two voxels are often placed close to the boundary of the vessel system. These do not capture any structure information. Thus, all values of the granulometry curve in front of the first minimum are cut off. The result is normalized and smoothed by fitting a cubic spline.

#### Intussusceptive pillar intensity

IP are tunnels in the vessel lumen with an inner diameter between 1 and 5 μm. For pillar detection, we use an automatic approach presented in [23] for investigating angiogenesis in the lung. The algorithm consists of two steps: First, holes with an inner diameter between 1 and 5 μm are marked. Afterwards, markers whose Euler characteristic is 0 are considered topologically equivalent to a torus and counted as a pillar, see Fig. 4a). As the topology of markers hitting the image boundary is not observable, classification of these objects is impossible. Hence, pillar intensities are estimated on a reduced window ensuring that all markers centered in this window are completely contained in the image (minus-sampling, [24]). We consider three normalizations of *N*, the absolute number of pillars in the ROI: $N_{{\text{V}}} = \frac{N}{V\left( W \right)},$ the mean number of pillars per unit tissue volume, $N_{{{\text{V}},2}} = \frac{N}{V\left( X \right)},$ the mean number of pillars per unit microvascular volume, and $N_{{\text{L}}} = \frac{N}{{L_{{\text{V}}} V\left( W \right)}},$ the mean number of pillars per unit microvascular length.

## Results

### Microvascular morphometry: volume, length, and diameter

**Fig. 3:**
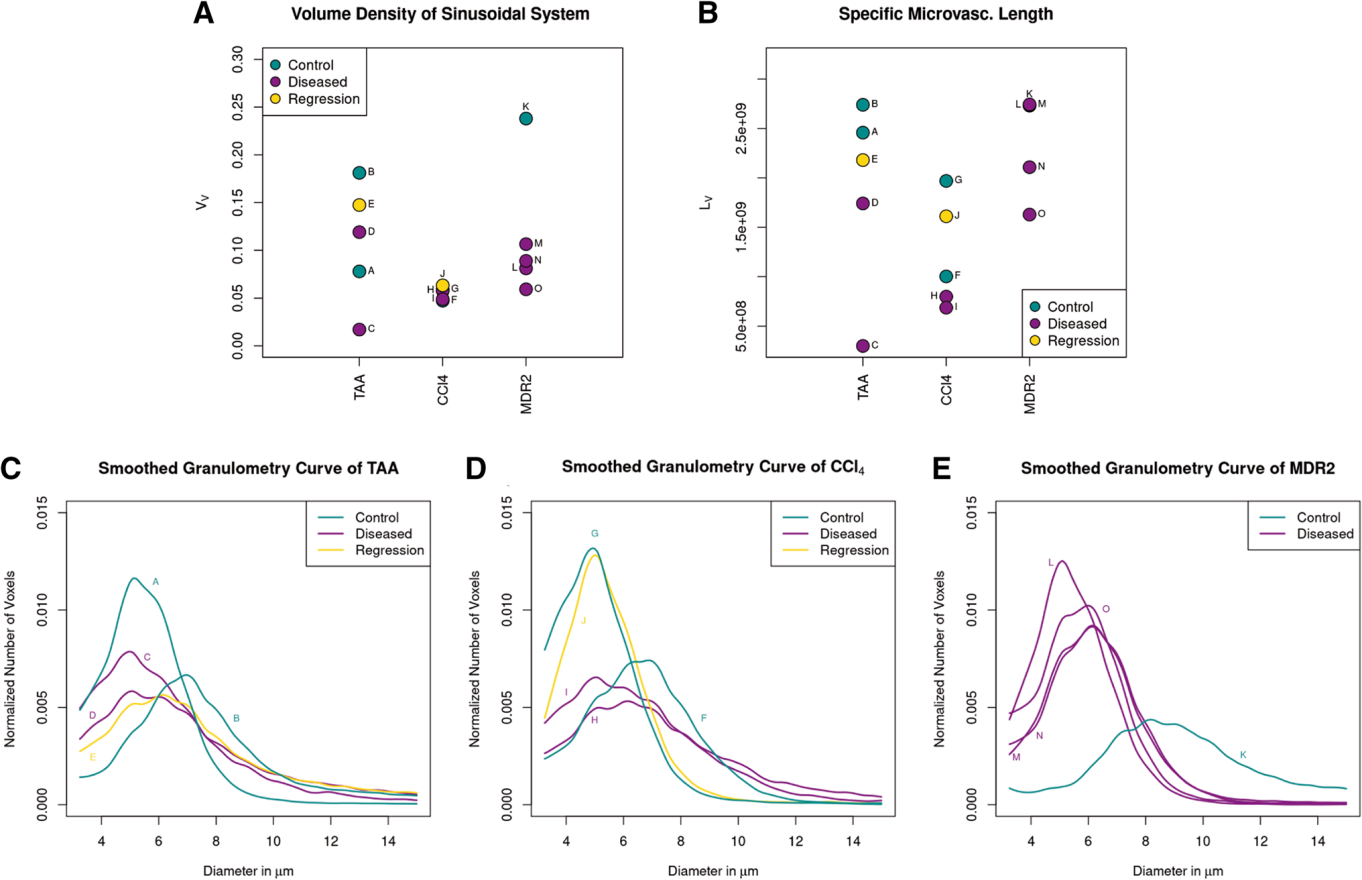
**a**, **b** Microvascular volume density and specific microvascular length for the three groups. **c**–**e** Granulometry curves

The volume densities and specific microvascular lengths for all scans as well as the corresponding granulometry curves are shown in Fig. 3. Microvascular volume density is reduced in the parenchchymal CCl4 fibrotic animals, while TAA fibrotic animals do not show a clear trend in microvascular volume density changes. Specific microvascular length is reduced in both TAA and CCl4 fibrotic animals with a trend towards normalization in the (mildly) regressed groups. Fibrotic and mildly regressed specimens in the TAA group show similar granulometry curves. A relevant difference in distribution is observed in two control specimens, one with smaller and one with larger diameters than in the disease group. Similar trends are observed for the CCl4 treatment group. In fibrotic specimens, a larger fraction of large vessel diameters is observed with a trend to normalization in the regression group.

In Mdr2KO biliary mice, the microvascular volume density is reduced in the fibrotic specimens. The specific microvascular lengths in the non-fibrotic control sample and two of the fibrotic specimens almost coincide. The other two specimens from the fibrotic group show a decline in specific microvascular length. The microvessel diameters are reduced in the fibrotic mice showing a reverse trend when compared to the hepatotoxin treatment groups.

### Intussusceptive pillar intensities

**Fig. 4:**
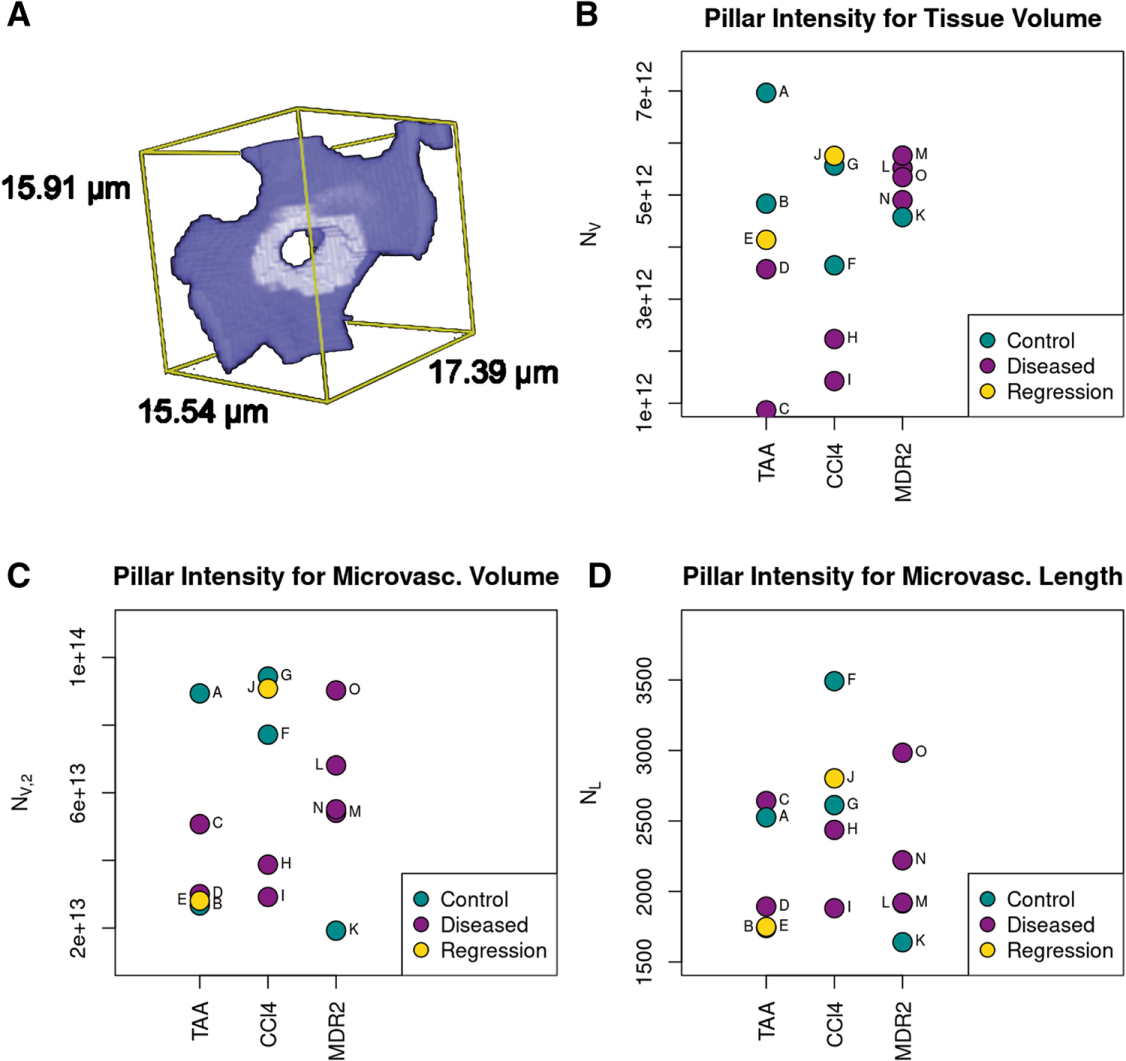
**a** Visualization of an intussusceptive pillar. **b**–**d** Pillar intensities in three different normalizations

Figure 4 summarizes the pillar intensities in the three different normalizations. In the two hepatotoxin groups with parenchymal fibrosis, the number of pillars per tissue volume is reduced vs the non-fibrotic controls. In the Mdr2KO group, fibrotic samples show higher pillar intensities than the control group. Similarly, pillar intensities w.r.t. microvascular volume show a decrease in the CCl4 and an increase in the Mdr2KO group. TAA does not show a clear trend due to the different diameter distributions in the two control specimens. However, a comparable trend can be observed for pillar intensities with respect to microvascular length. Fibrotic CCl4 livers show a decrease and fibrotic Mdr2KO livers an increase in pillar intensity. Again, there is no clear trend in TAA fibrotic mice.

## Discussion

In this proof-of-concept study, we assessed microvascular changes in three complementary murine liver fibrosis models using vascular corrosion casting and 3D imaging at a voxel edge length of 325 nm. Consequently, even the smallest sinusoidal and capillary vessels are resolved in the SRμCT data. On the other hand, only relatively small volumes of interest can be scanned which do not allow for a segmentation of the entire vascularity. This may incur a sampling variability especially in the biliary fibrotic mice that, as in human biliary fibrosis, display a more patchy distribution of fibrotic foci than the mice with parenchymal fibrosis due to the toxins CCl4 and TAA. Since the imaging technique is inherently non-destructive, a correlative imaging approach complementing our analysis with low resolution scans of the entire organ cast would be possible. Some trends in the morphological characteristics could be observed, although the small number of specimens examined in this study does not allow for final, statistically valid conclusions. We attribute the observed trends to remodeling processes in the development of hepatic fibrosis such as sinusoidal capillarization, i.e., a transformation of fenestrated hepatic sinusoids into closed capillaries. This process is characterized by an underlying collagenization of the spaces of Disse, as revealed morphologically by electron microscopy and functionally by multiple indicator dilution patterns [26]. Our results are not immediately comparable to the results from Peeters et al. [4] which is due to differences in the experimental setup (sample preparation, image acquisition, mode and duration of fibrosis induction). While we consider our models of hepatic fibrosis as highly robust and reproducible [22, 27, 28]. Therefore, our image analysis (evaluation per branch or per volume), and our inability to fully distinguish regenerative nodules and vascular septa need to be refined in future studies, including the definition of a comprehensive scale spanning analysis protocol.

The pillar intensities showed differences between the controls and Mdr2KO mice on the one hand and CCl4 and TAA mice on the other. This appears reasonable as the pillar intensity reflects the morphology of the capillary structure, especially in the intralobular effects,whereas the fibrotic changes in the Mdr2KO model are predominantly seen in the periportal region. To the best of our knowledge this is the first work investigating the 3D assessment of intussusceptive angiogenesis in hepatic fibrosis. In summary, we provide in this proof-of-concept study an approach for capturing microvascular alterations on the sinusoidal and capillary scale in hepatic fibrosis with evidence of features of intussusceptive angiogenesis. Our findings suggest that the formation of IP can differ greatly between different models of liver fibrosis. Further work is necessary to illuminate the dynamic interactions between structural adaptations of the hepatic microvascular architecture, functional parameters such as portal hypertension, and reversibility of advanced fibrosis.
